# Evaluation of radicular fracture resistance of maxillary premolars following non-surgical retreatment using two novel retreatment kits

**DOI:** 10.1186/s12903-026-09151-3

**Published:** 2026-07-14

**Authors:** Fatma M. Abu Naeem, Shaimaa Ibrahim Bakry, Ahmed S. ElSheshtawy, Ahmed Abdou, Hajer M. Abd ElHamid

**Affiliations:** 1 Department of Endodontics, Faculty of dentistry, Cairo University, Cairo, Egypt; 2 Department of Restorative Dentistry, Faculty of dentistry, Universiti Malaya, Kuala Lumpur, Malaysia; 3 Department of Endodontics, Faculty of dentistry, Modern University for Technology and Information, Cairo, Egypt

**Keywords:** Retreatment, Maxillary premolar, Instrumentation, Fracture resistance, Retreaty, Xp endo Rise

## Abstract

**Background:**

The structural integrity of teeth after endodontic retreatment depends on the amount of remaining dentin. Excessive instrumentation can weaken roots and predispose them to fracture. This study evaluated the fracture resistance of maxillary premolars following non-surgical retreatment (NS-ReT) with Retreaty and XP-endo Rise systems.

**Materials and methods:**

Forty-eight maxillary first premolar roots were randomly assigned to 4 groups (*n* = 12); G1-NC (Negative control, non-instrumented), G2-PC (Positive control, instrumented, not obturated), G3-RETY (Retreaty), and G4-XPER (XP-endo Rise). G2, G3 and G4 groups were prepared using the iRace system. Groups G3 and G4 were obturated with gutta-percha and resin sealer. Gutta percha removal was performed using Retreaty (G3-RETY) and XP-endo Rise retreatment files (G4-XPER). All samples were subjected to fracture testing using a universal testing machine & the maximum fracture load (N) was recorded. Gutta percha removal times (Tf) and total time of retreatment were also measured. Statistical analysis was performed using a mixed-model analysis of variance (ANOVA) (α = 0.05).

**Results:**

The fracture resistance of G1-NC (942.92 ± 24.73 N) had significantly higher values compared to all other groups. Fracture values for the G2-PC (829.62 ± 71.55 N) and the G3-RETY (818.29 ± 73.14 N) did not significantly differ from each other (*p* = 1.000), but both were significantly higher than the G4-XPER (489.37 ± 33.47 N). The total time of retreatment was significantly reduced when using the G3-RETY (3.89 ± 0.84 min) compared to the G4-XPER (13.30 ± 2.05 min), *p* < .001.

**Conclusions:**

Teeth retreated using the Retreaty instruments demonstrated superior fracture resistance compared to XP-endo Rise and their values were comparable to the positive control group. Moreover, Retreaty file system showed faster gutta-percha removal and canal preparation during retreatments.

## Introduction

Apical periodontitis following primary endodontic treatment may occur due to the presence of residual necrotic tissues, bacteria or percolation across the root canal system into the periapical tissues. Bacterial persistence after primary root canal treatment is a major cause of post-treatment apical periodontitis. These persistent microorganisms are often organized as biofilms within the apical third of the root canal system. Their persistence is usually associated with technical shortcomings, such as inadequate disinfection or the absence of a proper coronal and/or apical seal [1]. Therefore, the main objective of non-surgical endodontic retreatment (NS-ReT) is to treat, and prevent recurrence of, post-treatment apical periodontitis by controlling causative factors of the disease. This can be achieved through disassembling all intra-canal filling materials, regaining patency across the apical foramen for proper disinfection with minimal loss of radicular structure, which will impact the long-term survival of the root canal treated tooth [2, 3]. Given the probability of more dentine removal during NS-ReT than during primary treatments, an endodontically retreated tooth is subjected to an 8- to 12-fold higher risk of developing root fractures than a tooth with a primary treatment [4]. Therefore, complete removal of the intra-canal filling materials, without inducing iatrogenic alterations, during NS-ReT improves access to the root canal spaces permitting thorough disinfection, and contributing to successful outcomes of NS-ReT.

Despite the presence of a wide range of root filling materials, such as, silver points, gutta percha, resin-based materials as well as Hydraulic calcium silicate (HCSB) based materials, gutta percha remains the most widely used obturation material, which is always used in combination with a variety of sealers types [5, 6]. Removal of intra-canal filling materials depends on many variables, for instance; the obturation technique, the type of the material(s) used and more importantly the root canal anatomy. In this context, removal of a single cone obturation is relatively straightforward when compared to removing a well-compacted obturation performed with cold lateral compaction or warm obturation technique [5].

Being a major part of NS-ReT, gutta percha disassembly is usually performed using mechanical methods, utilizing hand and/or rotary instruments [7, 8]. Such mechanical methods may be combined with chemical solutions “solvents” or ultrasonic tools to facilitate complete removal of materials from the retreated root canal space.

Numerous nickel titanium rotary instruments have been introduced to facilitate gutta percha removal during NS-ReT and significant modifications have been made to improve their clinical performance. such modifications involved metallurgical modifications through proprietary heat treatments as well as design modifications, aiming at increasing the instruments’ cutting efficiency, fracture resistance while preserving as much radicular structure as possible [7–9]. 

Maxillary premolars have a unique anatomy since they have narrow mesiodistal dimensions, steep cuspal inclines as well as developmental root concavities. Such anatomical features make such teeth prone to mechanical failures, especially with extensive loss of coronal and/or radicular structure following endodontic treatment and/or retreatment. Moreover, their position in the dental arch, bears a risk for vertical fractures more than other teeth [10–12]. Such anatomical features necessitate adopting a minimally invasive approach during endodontic re-intervention, to enhance their long-term survival [13]. 

XP-endo Rise retreatment instruments (XPER) (FKG Dentaire Sàrl, La Chaux-de-Fonds, Switzerland) were introduced in 2022, as a descendant of the XP-endo instruments. Chronologically, in 2015, the first instrument introduced was the XP-endo Finisher (FKG Dentaire Sàrl, La Chaux-de-Fonds, Switzerland) and was manufactured from a MAX-wire alloy utilizing martensite-austenite electropolish flex technology which combine shape memory properties with super-elasticity for safe and efficient radicular instrumentation. In 2016, the manufacturer introduced the XP-endo Shaper, followed by XP-endo retreatment line in 2017 [14]. According to the manufacturer, the novelty in XPER files is the modified tip design having 6 facets instead of 3 in previous system generations. Moreover, the stiffer alloy and the modified tip design improves the effectiveness of removal of intra-canal filling materials [14, 15]. Due to their unique snake-like design, XPER instruments have the ability to expand beyond its original core (ISO 27 tip, 0.01 taper) to a size of ISO 30 with 0.04 taper. This behavior of the instrument allows it to address the 3-dimensional nature of the root canal space, while reaching most of the materials within the root canal in significantly less preparation time.

Retreaty (RETY) (Dental Perfect, Shenzhen, China) is another recently introduced retreatment instrument set designed to perform gutta-percha disassembly and reshaping of the root canal system during NS-ReT. RETY is a hybrid instrument set, composed of 5 instruments manufactured with 3 different heat treatments. The incorporation of files with different heat treatments in the same instrument set aims to address the anatomical variations in different parts of the root canal system. Moreover, RETY instruments have a unified cross-sectional design, an italic S-shaped cross-section. The manufacturer claims that the combination of different heat treatments with a unique and unified cross-sectional design, aims at efficient removal of most of the materials inside the root canal system, in a considerably less chairside time (https://www.dental-perfect.com/download) [16, 17]. 

To the best of the authors’ knowledge, no previous studies in the literature compared the fracture resistance of maxillary premolar roots following retreatment using XP-endo Rise retreatment versus using Retreaty instruments. Therefore, the aim of this study was to evaluate the radicular fracture resistance of maxillary premolars following retreatment using XP-endo Rise and Retreaty instrument and to calculate the time needed to complete gutta percha disassembly. The null hypotheses tested were that no difference would be found in terms of fracture resistance in maxillary premolar roots after using XPER or RETY, and that there would be no difference in the time needed to complete gutta percha disassembly and retreatment time using both instrument sets.

## Materials and methods

**Fig. 1:**
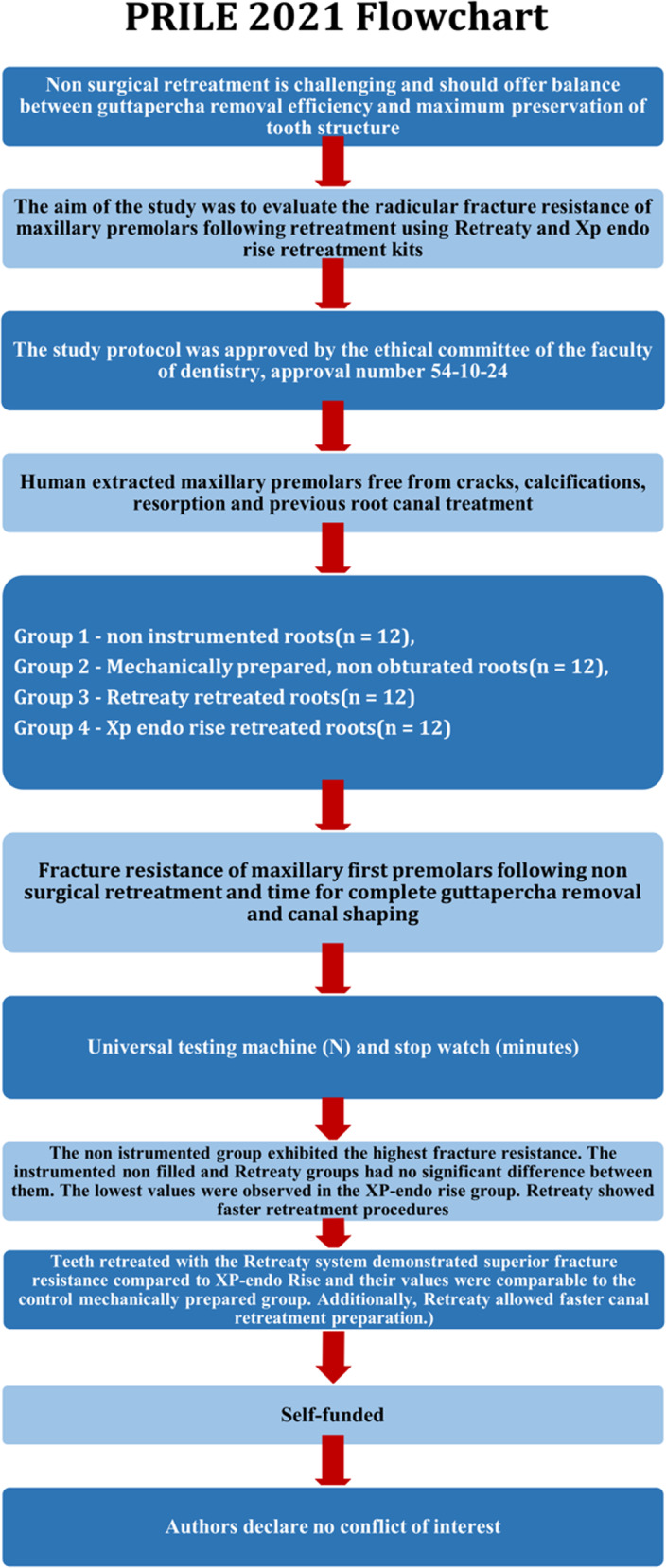
PRILE 2021 flow chart for the study

The study protocol received approval from the institutional ethics committee (approval number 54-10-24). This laboratory study was prepared in accordance with the Preferred Reporting Items for Laboratory Studies in Endodontology (PRILE) 2021 guidelines (Fig. 1).

### Sample size calculation

A power analysis performed using variance analysis in G*Power 3.1 (Heinrich Heine University, Düsseldorf, Germany). The expected effect size (f = 2.88) was calculated based on group means and a pooled standard deviation (σ = 39.96) [3], To detect this effect with an alpha error probability of 0.05 and a statistical power of 95% across 4 groups, the analysis indicated a minimum required total sample size of 8 specimens (*n* = 2, each group). To enhance statistical reliability and buffer against biological variation, the final sample size was purposefully increased to 24 premolars (*n* = 6 per group, 48 total roots). While this initial sample size estimation modeled a single-rooted tooth as the primary experimental unit, the potential clustering effect of evaluating premolar teeth with multiple roots was mathematically accounted for in the final statistical approach. Specifically, the employment of a mixed-model repeated-measures ANOVA, combined with tripling the minimum required sample size, ensured robust statistical power.

### Sample selection

After approval of the institutional research ethics committee, twenty-four maxillary first premolars (48 roots)-that were extracted due to periodontal or orthodontic reasons-were selected for this study from a pool of extracted teeth, at the oral and maxillofacial department in faculty of dentistry, Cairo university.

Teeth were selected so that each tooth have a total length of 20–22 mm and with straight buccal and palatal root (< 10° curvature, based on schneider’s radiographic technique) [4, 18]. Moreover, teeth were selected to have widely separated roots and fully formed apices. Digital radiographs, with mesiodistal and buccolingual projections were taken prior to sectioning, randomization and group allocation, to exclude teeth with calcified canals, or internal resorption. Teeth having external visible root defects, such as cracks, were excluded and replaced from the pre-mentioned pool.

The diameter of the root at the cemento-enamel junction (CEJ) was measured from both the mesiodistal and buccolingual aspects using a digital caliper. The diameters buccolingually and mesiodistally were ranging between 6 and 8 mm and 4–5.5 mm, respectively [19].

### Specimens’ preparation, randomization and grouping

The selected teeth were immersed in 5.25% NaOCl (Clorox; Nobel wax factories for Chemicals, Cairo, Egypt) solution for one minute, then stored in sterile saline container until use in the study. A computer-generated random sequence (https://www.random.org) was used for group allocation, concealed in numbered, sealed, opaque containers. Randomization was performed by an investigator/author who is not involved in the endodontic procedures (S.I.B), and both the outcome assessor (F.M.A) and the statistician (A.A.) were blinded to the group allocations.

Teeth were decoronated at or just below the CEJ, while maintaining the furcation area, to standardize specimens’ length to 14 mm. Thereafter, decoronated specimens were sectioned through the furcation using a diamond disc (Abrasive technology, Canada, Lewis Center, Ohio) under copious water cooling yielding a separate buccal and palatal root from each specimen.

Specimens were then randomly allocated into 4 groups (*n* = 6 teeth, each group = 12 roots), as follows;

- G1-NC: Negative control, non-instrumented and non-obturated
- G2-PC: Positive control, instrumented and not obturated
- G3-RTY: Instrumented, obturated and retreated using Retreaty files
- G4-XPER: Instrumented, obturated and retreated using XP-endo Rise retreatment files

### Endodontic procedures

A single and experienced endodontic operator performed all the treatment and retreatment procedures (A.S.E).

Roots in groups G2-PC, G3-RETY, G4-XPER, were prepared using iRace system (FKG Dentaire Sàrl, La Chaux-de-Fonds, Switzerland) apical patency was performed and secured using a # 10 K-file (Dentsply Sirona, PA, York). The working length was adjusted to 1 mm short of the apical foramen. The iRace instruments were used in X-smart plus endodontic motor (Dentsply Sirona, Switzerland, Ballaigues) according to the manufacturer’s recommendation (speed = 600 rpm, torque = 1.5 N.cm). canal preparation sequence involved coronal third preparation using R1 file (#15/0.06) followed by full length preparation by R2 (25/0.04) and finishing the preparation to R3 file (30/0.04). Canals were irrigated with 2 mL of 2.5% NaOCl (Clorox; Nobel wax factories for Chemicals, Cairo, Egypt) between each 2 successive files. afterwards, canals were irrigated using 2 mL of 17% ethylenediaminetetraacetic acid (EDTA; META BIOMED co.ltd, Cheongju, Korea) followed by 2 mL of 2.5% NaOCl then final rinsing with 10 ml distilled water. Afterwards, canals were dried using size matching paper points (META BIOMED co.ltd, Cheongju, Korea).

In the intervention groups (G3-RETY and G4-XPER), obturation was done using gutta percha (META BIOMED co.ltd, Cheongju, Korea) and epoxy resin-based sealer (ADseal; META BIOMED co. ltd, Cheongju, Korea), dispensed through a 13.5 g auto-mix syringe. The selected master gutta percha point (30/0.04) was coated with the sealer and inserted to the full working length. Accessory gutta percha cones (#25/0.02) were laterally compacted using a finger spreader (#25/0.02) (Mani, Inc, Japan) to complete the obturation procedures. Thereafter, a heat carrier was used to remove excess gutta percha at the level of the root canal orifice. Roots were then radiographed from mesiodistal and buccolingual aspects to confirm good quality of the obturation phase. The canal orifice was sealed using a provisional material (Coltosol F, Coltene, Altstätten, Germany). The obturated specimens were stored in 100% humidity and at 37 °C for 1 month to ensure the complete setting of the sealer.

### Retreatment procedures

All the root specimens of both intervention groups (G3-RETY and G4-XPER) were placed in temperature-controlled water bath at 37 °C, prior to initiation of retreatment procedures to simulate clinical in-vivo conditions. Similarly, all irrigation syringes were kept in a 37 °C water bath throughout the retreatment phases [15, 20].

Before initiation of retreatment, and following the manufacturer’s recommendations in protocol cards, one drop of solvent (Endosolv; Septodont, Saint-Maur-Des-Fosses, Cedex France) was placed into the canal orifice using the graduated dropper provided by the manufacturer, and was left for 1 min to facilitate penetration of the root filling by the introductory files of the tested instruments (DR1 file (#30/0.10) in XPER system, and the Bull-Y file (#25/0.07) in RETY system). No solvent has been beyond the coronal third during the retreatment procedures.

### Group 3 (G3-RETY)

Retreaty files (RETY) (Dental Perfect, Shenzhen, China) were used in an X-smart Plus endodontic motor according to manufacturer’s recommendation (torque = 2.5 N.cm, speed = 450 rpm). Gutta removal from the coronal third (coronal 4 mm) of the root canal was performed using Bull-Y (25 apical size, 0.07 taper). Coronal 2/3 gutta percha evacuation was performed using Skinn-Y #25 apical size, 0.04 taper) for coronal 2/3 of the root in a gentle pecking maneuver of 1–3 mm vertical strokes. The apical third is negotiated first using a stainless-steel hand file (#15, Mani, Inc, Japan). Once patency was achieved, full length gutta-percha removal was done consecutively using Shap-Y1 (20/0.05), followed by Shap-Y2 (25/0.05). Final apical enlargement was performed using Shap-Y3 (#30/0.05). Irrigation was performed using 2.5% NaOCl in between files, throughout the retreatment procedures. Complete Gutta-percha removal was considered successful when no more gutta-percha or sealer debris could be visualized between the flutes of the last instrument. Complete gutta percha removal was additionally confirmed radiographically.

### Group 4 (G4-XPER)

XP-endo Rise retreatment instruments (XPER) (FKG Dentaire Sàrl, La Chaux-de-Fonds, Switzerland) were used in an X-smart plus endodontic motor according to manufacturer’s recommended torque and speed settings. DR1 file (#30/0.10, torque = 1.5 N.cm, speed = 1000 rpm) was used for coronal third (coronal 4 mm), followed by XPER shaper (#30/0.04, torque = 1 N.cm, speed = 1000 rpm) for middle and apical thirds, in gentle pecking strokes, until the working length is reached. If the XPER shaper failed to reach the working length after 5 strokes, the file withdrawn, canal is irrigated with 2 ml, 2.5% NaOCl for 30 s then the procedure is repeated until reaching the full working length [20]. XPER finisher R (#30/0.00, torque = 1 N.cm, speed = 800 rpm) was used for finishing the retreatment procedure. Gutta-percha removal was judged successful according to the same criteria followed in G3-RETY group.

### Filling removal time (Tf)

The total filling removal time (Tf) was measured from the introduction of the first instrument into the canal until withdrawal of the last instrument. A stopwatch was used to record T1, the time required to reach the working length (WL), and T2, the time to complete filling removal. Tf was calculated as the summation of T1 and T2, excluding the time spent on instrument changes and irrigation [4].

### Fracture resistance testing

**Fig. 2:**
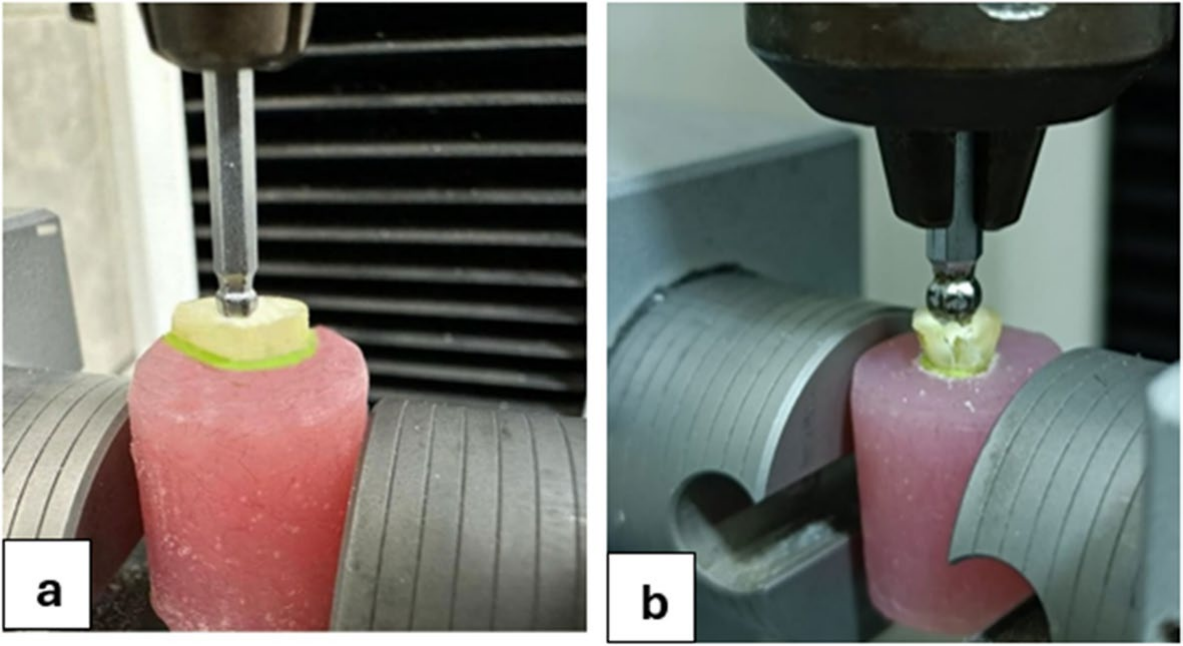
Fracture resistance test of specimens using universal testing machine. **a** Load application at the center of the root, (**b**) Specimen after fracture

Root specimens were coated with a thin layer of polyvinylsiloxane impression material (Elite HD+, Zhermack SpA, Badia Polesine, Italy) then they were vertically placed inside an acrylic resin mold (Acrostone dental products, Cairo, Egypt) [21]. Roots in their artificial sockets were mounted in a 5 kN universal testing machine (Instron 3345 Series, Wycombe, UK). continuous, static and vertical compressive load was applied at the center of the canal orifices using a 3 mm round ended spherical tip placed axially over the root canal orifice and touching the mesial and distal aspects of the root to simulate forces from an antagonist tooth. The load was applied along the long axis of the root specimen with a loading rate of 1 mm/min until fracture [22, 23]. The load at which failure occurred was recorded in Newtons (N). (Fig. 2)

### Statistical analysis

Data was analyzed using IBM SPSS Statistics (V23, Armonk, NY, USA). Normality was assessed with Shapiro-Wilk tests, which indicated a normal data distribution. To evaluate the effects of the treatment group and root location on fracture resistance, a mixed-model analysis of variance (MANOVA) was conducted. The model included the treatment group (G1-NC, G2-PC, G3-RETY, and G4-XPER) as the between-subjects factor and root location (Buccal, Palatal) as the within-subjects repeated measure. The assumption of sphericity for the within-subjects factor was evaluated using Mauchly’s test. Post-hoc pairwise comparisons to evaluate significant main effects were adjusted using the Bonferroni correction (α = 0.05). By executing a Within-Subjects Repeated Measures ANOVA (with the individual tooth as the subject and the root type as the within-subject factor), the model successfully partitions out the intra-tooth baseline covariance. Moreover, it prevents pseudo replication while accurately reflecting the true sample architecture (*n* = 24 teeth, generating 48 observational root measurements).

## Results

### Fracture resistance

**Fig. 3:**
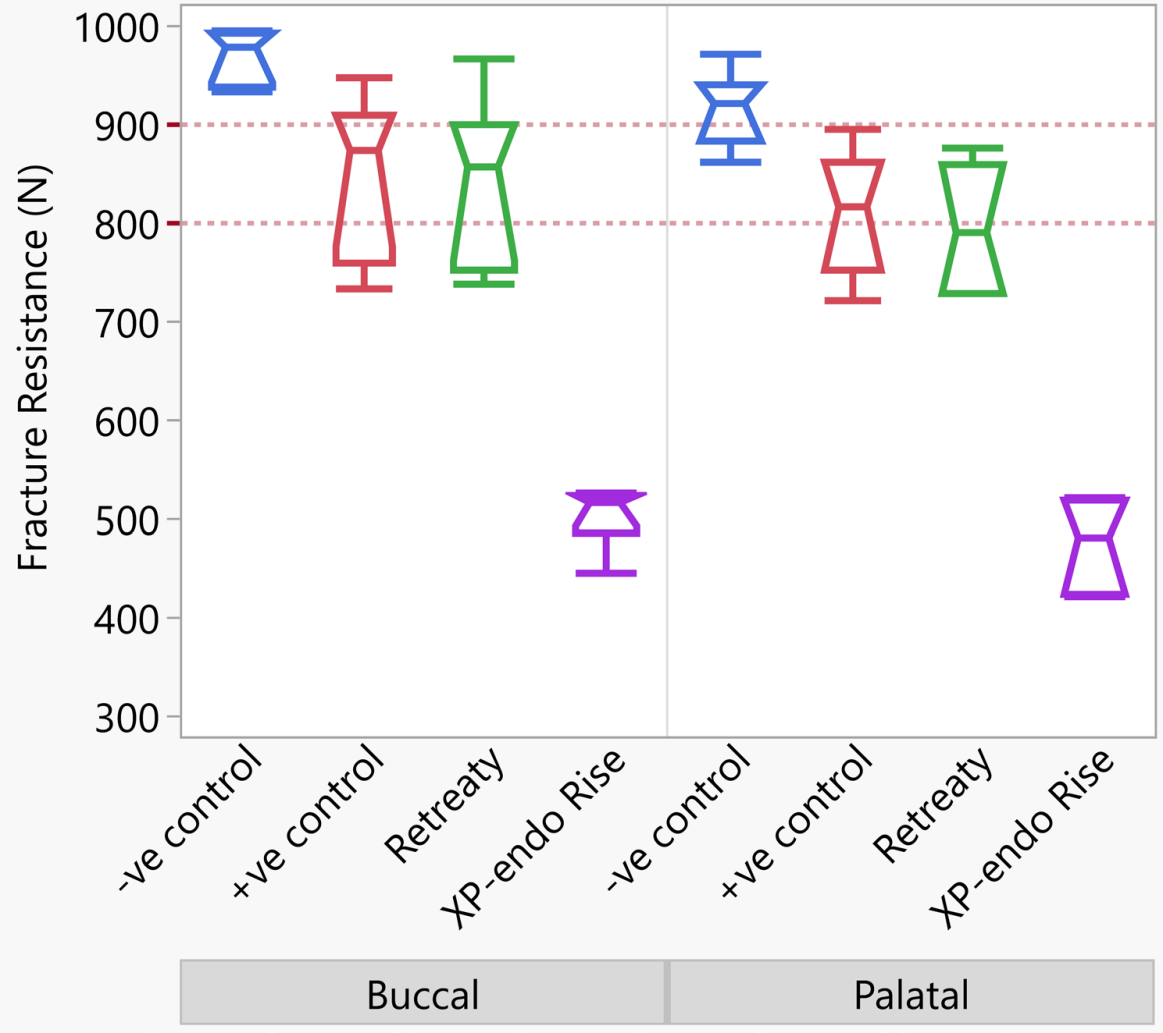
Box plots display the fracture resistance (N) at the Buccal and Palatal roots. Dashed red lines at 800 N and 900 N are provided to aid visual comparison of the values between the two root locations. G3-RETY group showed similar results to G2-PC group (instrumented, non-obturated) while G4-XPER group showed a lower fracture resistance values compared to all groups

The fracture resistance data values for all groups are presented in Table 1, Fig. 3. The assumption of sphericity was not applicable as the root location was only 2 levels so there was no need for correction, Mauchly’s W = 1.000. The main effect of the different root location was statistically significant, *F* (1, 20) = 21.40, *p* <.001, η = 0.517. Pairwise comparisons indicated that fracture measures were significantly higher in the Buccal root location (791.19 ± 186.23 N) compared to the Palatal root location (748.91 ± 176.17 N), representing a mean difference of 42.27, *p* <.001.

**Table 1:** Mean ± standard deviation [95% confidence interval] and pairwise Comparisons of Fracture resistance (N) for different groups and root Location

Group	Buccal	Palatal	Effect size	*p*-value	overall main (Buccal+Palatal)
G1-NC	969.9^a^ ± 27.34 [916.70, 1022.99]	915.99^a^±37.54 [870.06, 961.92]	0.303	0.008	942.92^a^±24.73 [897.05, 988.78]
G2-PC	848.7^b^ ± 82.24 [795.56, 901.85]	810.54^b^±62.6 [764.61, 856.46]	0.179	0.05	829.62^b^±71.55 [783.75, 875.49]
G3-RETY	841.7^b^ ± 84.26 [788.55, 894.84]	794.89^b^±63.41 [748.96, 840.82]	0.247	0.019	818.29^b^±73.14 [772.43, 864.16]
G4-XPER	504.5^c^ ± 31.15 [451.35, 557.65]	474.23^c^±47.82 [428.30, 520.16]	0.121	0.113	489.37^c^±33.47 [443.50, 535.23]
Effect size	0.902	0.919			0.922
*p*-value	< 0.001	< 0.001			< 0.001

Different superscript letters (a, b, c) within the same column indicate statistically significant differences between treatment groups based on post hoc comparisons (*p* <.05). The *p*-values and effect sizes (measured as partial eta-squared) located in the rightmost columns represent the main effect comparing the Buccal and Palatal locations within each specific treatment group. The *p*-values and effect sizes in the bottom rows represent the main effect of the treatment group within each specific root location, as well as the overall main effect for the average values

Moreover, there was a statistically significant main effect of the different treatment groups, *F* (3, 20) = 78.98, *p* <.001, η = 0.922. Post hoc analyses using the Bonferroni correction revealed that the G1-NC group (942.92 ± 24.73 N) had significantly higher fracture resistance values than all other groups [G2-PC group (*p* =.010), G3-RETY group (*p* =.004), and G4-XPER group (*p* <.001)]. Fracture resistance values for the G2-PC group (829.62 ± 71.55 N) and the G3-RETY group (818.29 ± 73.14 N) did not significantly differ from each other (*p* = 1.000), but both were significantly higher than the G4-XPER group (489.37 ± 33.47 N).

The interaction effect between root location and treatment group was not statistically significant, *F* (3, 20) = 0.32, *p* =.814, η = 0.045, suggesting that the difference in fracture between the buccal and palatal roots remained consistent across all four treatment groups.

When evaluating the root locations separately, the same pattern of treatment effects was observed. Within both the buccal and palatal roots, the G1-NC group demonstrated significantly higher fracture resistance compared to all other groups (*p* <.001). The G2-PC and G3-RETY groups exhibited similar results, with no statistically significant difference between them (*p* = 1.000); however, both showed significantly higher fracture resistance than the G4-XPER group (*p* <.001).

Consequently, the G4-XPER group yielded -significantly- the lowest fracture resistance values among all treatments. Furthermore, when comparing the two root aspects within each specific treatment group, the buccal root consistently displayed higher fracture resistance than the palatal root. This anatomical difference was statistically significant for the G1-NC (*p* =.008), G2-PC (*p* =.050), and G3-RETY (*p* =.019) groups, but it was not statistically significant for the G4-XPER group (*p* =.113).

### Retreatment time

Analysis of the time needed to complete retreatment (Tf) revealed that the G3-RETY group required significantly less time to reach the working length (2.88 ± 0.76 min.) compared to the G4-XPER group (10.62 ± 1.81 min), *p* <.001. Similarly, the time required to complete filling removal was significantly shorter for the G3-RETY group (1.02 ± 0.28 min) than for the G4-XPER group (2.68 ± 1.09 min), *p* <.001. Consequently, the total time of retreatment was significantly reduced when using the Retreaty system (3.89 ± 0.84 min) compared to the XP-endo Rise system (13.30 ± 2.05 min), *p* <.001. The calculated effect sizes for all three retreatment phases far exceeded the standard threshold for a large effect (Cohen’s *d* ≥ 0.80). This indicates that the time-saving benefits of the Retreaty system, compared to the XP-endo Rise, are of substantial practical and clinical significance. The mean and standard deviation of the retreatment time intervals (minutes) of the two systems are presented in Table 2.

**Table 2:** Mean and SD of the Retreatment times (minutes) between G3-RETY and G4-XPER

Group	T1 Time required to reach the working length		T2 Time to complete filling removal		Tf Total time of retreatment	
	mean ± SD	min - max	mean ± SD	min - max	mean ± SD	min - max
G3-RETY	2.875 ± 0.757	2–4.16	1.016 ± 0.282	0.6–1.6	3.891 ± 0.835	2.6–5.3
G4-XPER	10.619 ± 1.811	8.3–15.7	2.68 ± 1.093	1.5- 5	13.301 ± 2.054	9.1–16.6
Effect size	5.58		2.9		6.00	
*p* value	< 0.001		< 0.001		< 0.001	

Effect sizes were calculated using Cohen’s d; all observed differences represent a large effect (d ≥ 0.80)

## Discussion

Post-treatment apical periodontitis, often referred to as post-treatment disease, is a disease occurring in previously endodontically treated teeth in which the treatment was of substandard quality in terms of infection control. Nevertheless, post-treatment apical periodontitis can also occur in teeth with apparently proper endodontic treatment [24–26]. Bacteria can endure the canal disinfection protocol, hence termed; persistent infection or might regain access to the root canal system due to coronal leakage, and therefore termed; secondary infection [1]. Despite the presence of non-microbial causes for post-treatment apical periodontitis such as; true cysts and foreign body reaction, microbial causes of treatment/retreatment failures remain the most common etiology for post-treatment apical periodontitis, as reported by several cultural and molecular microbiology studies [1, 24]. Therefore, Elimination of causes of such a disease, through Ns-ReT or surgical retreatment is mandatory to restore the endodontically treated/retreated tooth to a state of biological and functional health. In the same context, it is worth mentioning that extraction of teeth affected by post-treatment apical periodontitis is a valid therapeutic option, if the tooth is judged non-salvageable or in case the patient did not consent further attempts to save the tooth [1, 24, 26].

In the present study, maxillary premolar roots were selected, since it has been reported in literature that root fractures are the third most common cause of extraction of endodontically treated teeth, and mostly affecting maxillary premolars [27, 28]. Moreover, maxillary bifurcated premolars have peculiar anatomy, such as; the high convexity of their roots [29], the presence of furcation grooves on the palatal aspect of the buccal roots, with very thin dentinal walls at the groove area [30–32], as well as their position and shape in the dental arch [10] make such teeth are more prone to development of microcracks and/or vertical root fractures, especially with mechanical root canal instrumentation [31].

In the same context, teeth that undergo retreatment are therefore more susceptible to mechanical failures, such as vertical root fractures, with their fracture resistance often decreasing as more dentin is removed. Additionally, factors such as the instrumentation technique and instruments that are used for root canal filling removal, adversely affecting the tooth resistance to fracture [33–37]. In the present study, the tested specimens were coated with a thin layer of vinyl polysiloxane impression material to replicate the periodontal ligament. The bone support was simulated by placing the rubber-coated roots in polymethylmethacrylate resin mold. This method is an approach to mimic the natural movement of teeth under loading [21].

In a recent narrative review, the authors critically discussed the effect of preparation sizes of experimental models on investigating material removal by retreatment systems in research. They recommended a final apical preparation size to be larger or at least, of the same size as the final instrument in the retreatment system to be tested. Moreover, the Retreaty and XP-endo Rise Retreatment systems have similar final apical sizes, equivalent to ISO #30. Therefore, in this study, samples of G3-RETY and G4-XPER groups were prepared and obturated to an apical size of ISO #30. Such preparation size also correlated to proper penetration of irrigation and improves cleanliness of the apical third [38].

Results from previous studies showed that endodontically treated teeth have significantly reduced fracture resistance when prepared with greater taper instruments due to the magnified effect of stresses resulting from masticatory forces. Therefore, the Retreaty system (final Size taper #30/0.05) and XP-endo Rise retreatment system (final size/taper #30/0.04) were selected for comparative evaluation, since they have comparable final preparation sizes and limited tapers. This is in accordance with previous studies that reported increased fracture resistance of endodontically treated teeth with tapers less than 6% [35, 39].

This study compared Retreaty and XP-endo Rise Retreatment systems in terms of the effect on fracture resistance. The negative control group (G1-NC) showed significantly higher fracture resistance values (942.92 ± 24.73 N) compared to all other groups. This was in accordance with previous studies [22, 36, 37]. This can be attributed to the fact that unprepared and anatomically intact teeth, retain their structural integrity as well as their dentinal properties [28, 40].

Moreover, the results of our investigation showed that there was no statistical difference in the fracture resistance values between the positive control group (G2-PC) (829.62 ± 71.55 N) and the Retreaty group (G3-RETY: 818.29 ± 73.14 N), where both were significantly higher than the XP-endo Rise Retreatment group (G4-XPER: 489.37 ± 33.47 N). These results were in contrast to Bohra et al., 2025 [41] who reported that XP-endo Rise instruments showed the least reduction in fractures resistance compared to ProTaper Ultimate and JIZAI system, due to the less taper of XPER as well as its swaggering snake-like motion. on the other hand, our results can be in partial agreement with Moriya et al., 2023 [42] who reported that none of the canal instrumentation protocols were stress free but show varying level of stress concentration.

To the authors’ best knowledge, this is the first article to investigate the effect of using Retreaty system on the fracture resistance of endodontically treated teeth and there is only one article investigated the efficiency of the retreat system to remove filling material in comparison to Protaper Universal Retreatment system [17]. Our interpretation for the higher fracture resistance values of Retreaty (G3-RETY) group, may be due to the difference in size and taper between the initial instruments in both tested systems. The first instrument in the Retreaty system (Bull-Y, #25/0.07, 21 mm total length and 19 mm active working part), where the maximum flute diameter (MFD) of the instrument increase from D_0_ (#25) to a size of #53 at D_4_. On the other hand, the intro file for the XP-endo Rise Retreatment (DR1, #30/0.10, 21 mm total length and 15 mm active working part), where the MFD of the instrument increase from #30 at D_0_ to #70 at D_4_. Such difference in the MFD of the intro files might account for excessive dentin removal in the coronal parts of the root specimens, affecting ultimately their stress response to loading such interpretation, is in accordance with previous studies evaluating the effect of root canal preparation taper on the fracture resistance of endodontically treated and retreated teeth, especially with excessive coronal preparations [33, 43].

Our results revealed higher fracture resistance values of the buccal roots as compared to palatal roots in all the study groups, (G1-NC (*p* =.008), G2-PC (*p* =.050), and G3-RETY (*p* =.019). Only in the G4-XPER, there was not statistically significant difference in the fracture resistance values between the buccal and palatal roots (*p* =.113). Our results were in accordance with Laurentina et al., 2015 [44], who reported higher fracture resistance of buccal roots than palatal roots. However, these findings are contradictory to the assumption of fracture vulnerability of the buccal roots, owing to their unique anatomy, such as the high convexity, less buccal bone thickness and the furcation developmental grooves on the palatal aspects of the buccal roots [10, 29–31].

In literature, the investigations of the fracture resistance of buccal and palatal roots are sparse and inconsistent. The majority of studies evaluated the fracture resistance of the independent roots after restorative/prosthetic treatments [10, 45, 46]. Studies evaluating the effect of mechanical instrumentation during endodontic treatment or NS-ReT didn’t address the difference in fracture resistance values between the buccal and palatal roots [4, 22, 33, 36]. In this study, roots were the unit for fracture resistance testing instead of the whole tooth. This has been done by sectioning through the furcation, yielding a separate buccal and palatal root from each tooth. Such approach in the methodology aimed to eliminate the possible effect of any confounders [47]. 

Clinically, during centric occlusion, the palatal cusps are subjected to vertical/axial loading. This creates magnified stresses in the palatal cervical area. Since our results showed a lower fracture resistance of the palatal roots, the position of the occlusal contacts must be carefully designed in the post-endodontic restoration to minimize the effect of such stresses. İn this aspect, it is preferred to design a post-endodontic restoration with a 3-point occlusal loading (cusp-to-fossa) rather than a 4-point occlusal loading (cusp-to-marginal ridge) for a better stress distribution in endodontically treated teeth [48]. On the other hand, during lateral excursive movement, a maxillary premolar on a functioning side, the buccal cusp would be subjected to a lateral tipping forces as the lower premolar cusp slides along the palatal cusp inclines. This would create stress concentration in the buccal cervical area. To minimize the effect of such overload, acquisition of vertical ferrule of 1.5–2 mm is needed during post-endodontic restorative procedures. Moreover, placing the contact point on the bottom or middle of the buccal cusps, by reducing the cuspal inclined plane will help to reduce the stress concentration during lateral excursions [49]. In this context, it is of prime importance for the clinician to take into account such factors to improve the stress response of the endodontically treated tooth in order to enhance the long-term survival of the endodontically treated maxillary premolars.

In terms of post space preparation, this biomechanical variation introduces a compelling clinical paradox. Although the palatal root exhibits a lower fracture resistance, it is morphologically more favorable and predictable choice for post placement. The palatal canal is typically wider, straighter, and surrounded by sufficient bone bulk and more importantly lacks developmental depressions. Conversely, despite possessing higher fracture resistance, placing a post in the buccal root may bear a risk of an iatrogenic incident due to the presence of a developmental on its palatal surface significantly reduces the residual dentin thickness [10]. This architectural limitation substantially increases the risk of strip perforation or severe root weakening during post space preparation. Therefore, when a post is indicated for a bi-rooted maxillary premolar, clinicians should preferentially utilize the palatal canal [50]. In this context, it is of prime importance for the clinician to take into account such factors to improve the stress response of the endodontically treated tooth in order to enhance the long-term survival of the endodontically treated maxillary premolars.

Our study investigated the time needed to complete removal of intra-canal filling materials. This aimed to reflect the clinical efficiency of the tested systems as well as the chairside time needed for filling removal during NS-ReT. Moreover, the shorter the time needed to remove the filling, the more the time available to perform adequate disinfection of the root canal system, in attempts to improve the outcomes of NS-ReT [51] The results of the present study showed that shorter time is needed for the Retreaty instruments (G3-RETY) to desobturate the root canal as compared to XP-endo Rise Retreatment instruments (G4-XPER). These findings were in accordance with Alouda et al., 2025 [17], who reported that less time needed for the Retreaty instruments to reach the full working length compared to ProtTaper Universal Retreatment instruments. In literature, the description of the filling removal end-point is highly variable and inconsistent [5]. Several methods have proposed including, the ability of the instrument to reach the full length [52], absence of material remnants on the instruments’ flutes and in irrigating solutions [53], absence of materials on the root canal walls using magnification or radiographs [54, 55] as well as absence of dissolved materials on paper points “wicking”. In the present study, the filling removal was judged complete, when there were no material remnants visible on the last instruments’ flutes together with a clear irrigation free of any material debris. These were further confirmed by digital radiographs.

To facilitate penetration and removal of the filling material by the tested instruments, a solvent was used in the present study. This was in accordance with the methodologies followed in literature. However, the amount of solvent used doesn’t correlate to the efficiency of filling removal due to the inability to determine the actual amount of solvent that come into contact with filling material [5]. In our calculations of the total filling removal time (Tf), we excluded the time needed to change the instruments, clean instruments’ flutes and irrigation intervals. This is in accordance with previous studies that investigated the time needed to complete filling removal [5, 56, 57].

Based on the findings of our investigations, Retreaty instruments (G3-RETY, M = 2.875) reached the working length in significantly less time than the XP-endo Rise Retreatment instruments (G4-XPER, M = 10.619). On the other hand, instruments in G3-RETY group removed the root filling material completely in a significantly shorter time (M = 1.016), than instruments in G4-XPER (M = 2.68). To date, only one study evaluated the efficiency of retreat system to remove intra-canal filing materials [17].

Results of the present study demonstrated a statistically significant difference in the fracture resistance values and the time needed for gutta percha removal among the intervention groups, thus, the null hypothesis postulated in this study was rejected.

The present in vitro study was conducted in a controlled experimental setting; however, such settings are inevitably different from the complex biomechanical oral environment. This necessitates further investigation to more completely reflect clinical scenarios and achieve absolute clinical recommendations. For instance, this laboratory evaluation was conducted under static load. Future studies are necessary to fully validate these results under true in vivo conditions. However, these findings can serve as a foundation for future in vivo and clinical studies to validate the current observations. Furthermore, future studies assessing dentin removal, the smear layer, and the cleanliness of endodontically treated canals using advanced imaging modalities (e.g., micro-CT) can provide a comprehensive assessment of these new retreatment systems.

## Conclusion

Within the limitations of this laboratory study, it can be concluded that both the Retreaty and XP-endo Rise Retreatment systems can be used efficiently to remove intra-canal filling materials.

The Retreaty system caused less reduction in the fracture resistance than XP-endo Rise Retreatment system. Moreover, Retreaty file system showed significantly faster gutta-percha removal and canal preparation than XP-endo Rise Retreatment system.

## Data Availability

Data underlying the results of the present study may be obtained from the corresponding author upon reasonable request.

## Abbreviations

NS-ReT: Non-surgical retreatment
G1-NC: Group 1, Negative control
G2-PC: Group 2, Positive control
RETY: Retreaty instruments
XPER: XP-endo Rise Retreatment instruments
HCSB: Hydraulic calcium silicate-based materials
